# Venous Thromboembolism in Severe Burns Patients with Intravascular Warming Catheter: A Retrospective Cohort Study

**DOI:** 10.3390/ebj4010008

**Published:** 2023-02-12

**Authors:** Isabella Reid, Hadley Bortz, Aidan Burrell, Dashiell Gantner, Samara Rosenblum, Heather Cleland

**Affiliations:** 1 Victorian Adult Burns Unit, The Alfred Hospital, Melbourne, VIC 3004, Australia; 2 Department of Pharmacy, The Alfred Hospital, Melbourne, VIC 3004, Australia; 3 Department of Intensive Care, The Alfred Hospital, Melbourne, VIC 3004, Australia; 4 Australian and New Zealand Intensive Care Research Centre, Monash University, Melbourne, VIC 3000, Australia; 5 Department of Surgery, Central Clinical School, Monash University, Commercial Road, Melbourne, VIC 3000, Australia

**Keywords:** severe burns, hypothermia, intravascular temperature control, venous thromboembolism, burn surgery

## Abstract

*Background:* Use of intravascular warming catheters following major burns has been shown to be effective to maintain normothermia, but their use may be associated with complications. The aim of this study was to determine what proportion of patients with an intravascular warming catheter developed a potentially catheter-related venous thromboembolism (VTE) and to identify contributing risk factors. *Methods:* This was a retrospective cohort study of patients admitted to the Victorian Adult Burns Service January 2013 to July 2018 with major burns (TBSA > 20%) who had an ICY^TM^ intravascular warming catheter. Warming catheter insertion and other details were identified with a manual search of the patients’ medical records by a single author while incidence of VTE was determined by the coding department from a central database. *Results:* Forty patients had an intravascular warming catheter inserted during the study period. The number of patients in the catheter group that sustained a VTE was eight (20%), of which four (10%) could have been catheter-related due to the anatomical location. In the cases of the four potentially catheter-related VTE, other preventable VTE risk factors including suboptimal prophylactic anticoagulation (*n* = 2), prolonged catheter duration (*n* = 1) and prolonged haemoconcentration (*n* = 2) were identified. *Conclusions:* We found 20% of major burns patients with an intravascular warming device had significant VTE; however, only half of these may have been related to the catheter. A careful assessment for each patient that balances risks and benefits should be undertaken prior to using intravascular warming devices.

## 1. Introduction

Intravascular warming catheters are important as an effective prevention and treatment strategy for burns hypothermia, a serious complication of severe burns that is associated with increased mortality, coagulopathy, increased transfusion requirement, surgical site infection and delayed drug metabolism [1]. Burns hypothermia occurs as a result of the pathophysiology of severe burns and may be worsened by fluid resuscitation, long-distance referral to tertiary burns centres, administration of anaesthetic drugs, mechanical ventilation and intra-operative exposure for burns debridement [2,3]. Alternative warming strategies include a combination of warmed intravenous resuscitation fluids, increased ambient room temperature and forced hot air technologies; however, these are challenging to implement and less effective than intravascular warming catheters [4,5].

The ICY^TM^ intravascular warming device, a particular branded intravascular heat exchange catheter kit, was introduced at our institution in 2013; however, the safety of these intravascular warming catheters was brought into question after we observed a series of venous thromboembolism (VTE) in patients warmed with the catheter in our hospital. Shortly after this series of VTE events, the temperature management catheters were discontinued for use for all indications at our institution. There have been previous case reports and case series of warming-catheter-related VTE in burns and other critical illness, though the true associated risk remains unclear [6,7,8,9,10,11,12,13]. Irrespective of catheter use, major burns patients have long been recognised as a population inherently at high risk for VTE due to burn injury, inflammation, shock, prolonged immobilisation, use of intravenous lines and systemic hypercoagulability [14,15]. It is difficult to assess whether intravascular warming catheters pose an independent risk for VTE in addition to these other factors.

The aims of this study were to determine what proportion of patients with major burns who received an intravascular warming catheter developed a potentially catheter-related VTE and to identify contributing risk factors.

## 2. Materials and Methods

All patients with acute major burn injury (total body surface area affected (TBSA) >20%) admitted to the Victorian Adult Burns Service (VABS) from January 2013 to July 2018 whose data were entered in the Burns Registry of Australia and New Zealand (BRANZ) database were included in this study. The VABS is located at the Alfred Hospital, a major tertiary referral centre and a state-wide provider of care for all adults with complex or major burns injuries, and serves a population of 5.5 million in Melbourne, Australia. The BRANZ database routinely extracts details of all inpatient admissions to the VABS, including basic demographic data and burn severity indicators, in an automated non-biased manner. From 2013 to 2018, the ICY^TM^ intravascular warming catheter was used with the Thermogard^TM^ temperature management system, a computerised device that monitors and maintains a target temperature. The indications for intravascular temperature management included patients with major burns whose initial temperature was <35 °C and/or with an anticipated prolonged period in the operating theatre with risk of hypothermia (<35 °C). All ICY^TM^ catheters were inserted into the femoral vein as instructed by the manufacturer. All patients’ medical records were searched manually by a single investigator to identify intravascular warming catheter insertion. Incidence of VTE was determined by a member of the coding department unfamiliar with the study from a central database of coding discharge data. International Classification of Diseases (ICD-10) codes I26 (Pulmonary Embolism), I80 (Phlebitis and Thrombophlebitis) and I82 (Other Venous Embolism and Thrombosis) were used to identify cases of VTE. Individual patient medical records were subsequently reviewed by a single investigator to exclude cases that were deemed to be falsely classified as having deep vein thrombosis (DVT) or pulmonary embolism (PE), such as superficial thrombophlebitis. DVT and PE cases were defined as those with a confirmed radiological diagnosis from Doppler ultrasound, fluoroscopy or computed tomography pulmonary angiography (CTPA). To compare groups, Fisher’s exact test was used for nominal data and Welch’s *t*-test was used for interval and ratio level data with a significance level of *p* < 0.05.

This study was approved by the Alfred Health Research and Ethics Committee (Project number 244/20).

## 3. Results

**Figure 1:**
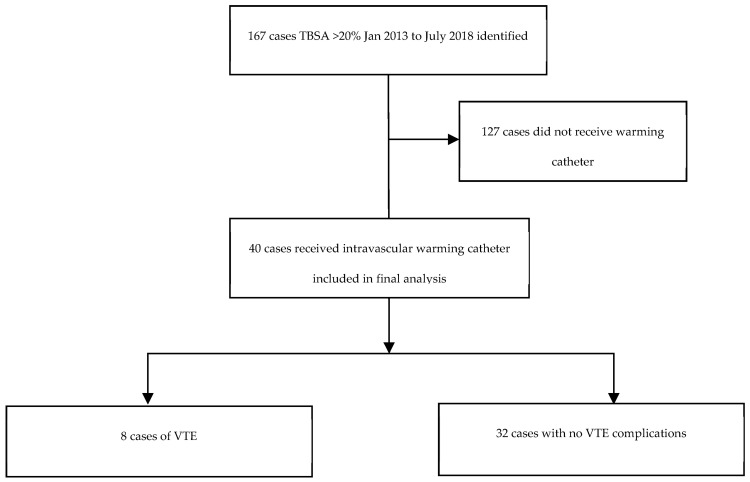
Cohort for analysis.

A total of 167 patients were admitted to the VABS with major burns during the study period (2013–2018). Within this group, 40 patients (24%) had an intravascular warming catheter inserted. The general characteristics of the catheter group compared to the non-catheter group are summarised in Table 1. The VTE rate in the catheter group was 20% (*n* = 8), whilst that in the non-catheter group was 4% (*n* = 5) (*p* < 0.01). Within the catheter group, nine cases of VTE were initially identified. One case was excluded as superficial thrombophlebitis; therefore, eight cases of VTE and thirty-two cases with no VTE complications were included in the final analysis (Figure 1).

For the cases of VTE in the intravascular warming catheter group, the median age was 49 (IQR 33–62) and the median TBSA was 60% (IQR 41–69) (Table 2). The patients were predominantly male (63%) and all required ICU admission and mechanical ventilation. One patient died due to multi-organ failure after 49 days in intensive care. There were six cases of DVT and two cases of PE. Of the DVTs identified, four cases were in the upper limb, one case was in the iliac vein extending into the IVC proximal to the warming catheter insertion site and one case was in the inferior vena cava (IVC). Therefore, four cases—the iliac DVT, the IVC thrombosis and the two PEs—were potentially warming-catheter-related. One of the cases of PE occurred immediately upon catheter removal. The patient went into cardiac arrest and, following successful cardiopulmonary resuscitation, echocardiography demonstrated global reduced ventricular function consistent with PE. CT pulmonary angiogram confirmed saddle PE. Subsequent embolectomy and IVC filter insertion was performed successfully. Four patients weighed 100 kg or more. The median catheter duration was four days (IQR 3,5). Prophylactic anticoagulation (enoxaparin 40 mg daily) was administered to 75% of patients (*n* = 6) and withheld for more than half or the entire duration of catheter insertion in two patients. Blood products were administered to 63% of patients (*n* = 5), and the median number of operations whilst the catheter was in situ was two. The median haematocrit and haemoglobin at the time of catheter insertion were 0.49 L/L and 161 g/L, respectively.

## 4. Discussion

This study represents the largest cohort of burns patients managed with an intravascular warming catheter in the literature. It aimed to determine the incidence of VTE in this cohort and identify other contributing risk factors. Burns hypothermia is a serious complication of severe burns, and therefore optimal management of this condition is critical. Intravascular warming catheters are a very effective strategy to treat this condition, as demonstrated by Prunet and colleagues, who reported significantly lower core body temperatures intra-operatively at 30 min intervals in major burns patients (TBSA > 40%) managed with intravascular warming catheters compared to traditional methods [5]. Therefore, it is important to elucidate whether these devices are safe to use.

The first finding of this study was that the patients who received an intravascular warming catheter had a significantly higher illness severity than those who did not (Table 1). The % TBSA, length of mechanical ventilation, length of ICU stay and total length of hospital stay were significantly higher in the catheter group compared to the non-catheter group. These are all recognised independent risk factors for VTE [16]. The VTE incidence for patients with an intravascular warming catheter (8/40, 20%) was significantly higher than that for the non-catheter group (5/127, 4%) (*p* < 0.01), as expected. Based on these confounders, it is impossible to make any conclusions about the comparative VTE incidence of these two groups due to the marked differences in baseline severity of illness.

The second finding of the study is that the overall VTE incidence for patients who received an intravascular warming catheter was 20%, whilst only 10% of VTE could possibly be attributed to the use of a warming catheter based on anatomical location. Of all the VTE cases recorded in the catheter group, four were DVTs in the upper limb, which were all related to central venous catheters previously inserted in the subclavian vein. The other four patients with VTE experienced PEs or DVTs in the pelvis, proximal lower limb or IVC (Table 2). As per product guidance, the warming catheters in this study were inserted exclusively into the femoral site. Therefore, the incidence of VTE that could have been directly related to the presence of the ICY^TM^ warming catheter was only 10% (4/40). This incidence is comparable to the overall VTE risk for severe burns patients reported in previous studies. Wait and colleagues, for example, reported a 7% incidence of symptomatic, lower limb DVT in ICU burns patients [17]. Furthermore, this incidence is comparable to the incidence of VTE associated with standard femoral venous catheters in ICU patients (10–22%) [11,18].

In the context of the previous lack of literature on the risk of VTE associated with warming catheters in burns, our study’s findings are informative. Two previous studies assessed the incidence of VTE in major burns cases with the use of a warming catheter and reported no VTE events; however, both studies were small and insufficiently powered. One was a retrospective case–control study of 23 burns patients (TBSA ≥ 25%) warmed with an intravascular catheter compared to traditional warming [4]. The second was a non-randomised controlled trial study of major burns patients (TBSA ≥ 40%) comparing four patients treated with intravascular warming to three patients treated with traditional warming and found no events of DVT [5]. Other studies have assessed the incidence rate of VTE in patients treated with intravascular temperature management devices for indications other than burns, including critical neurologic illness and out-of-hospital cardiac arrest. These studies have found varying rates of VTE. A non-randomised controlled trial of 296 patients with critical neurological injury observed a 3.3% and 7.8% VTE rate in the catheter and control groups, respectively [19]. A retrospective cohort study of 61 patients cooled for out of hospital cardiac arrest had a 14.7% rate of catheter-related thrombosis based on a combination of symptomatic VTE diagnosis and asymptomatic screening [10]. The incidence rate of VTE in our study is comparable to that of these other studies. Studies that have performed routine screening for VTE in patients with intravascular warming catheters have reported higher rates of VTE associated with intravascular warming devices. A retrospective review of 11 patients with severe head injuries who underwent intravascular cooling found a DVT rate of 50% with routine lower limb ultrasound post-removal of the catheter [9]. A further series of 25 patients who underwent therapeutic hypothermia after cardiac arrest reported a VTE rate of 25% based on bilateral lower limb ultrasound post-removal of the catheter [12]. It is possible that asymptomatic VTE cases in our burns cohort were underdiagnosed, thus posing a limitation to our study; nevertheless, the clinical significance of such unidentified asymptomatic DVT is not clear.

Further examination of the individual cases of VTE in patients who had a warming catheter inserted in this study highlighted preventable risk factors for VTE other than the use of warming catheter that could have led to this complication. First, four patients weighed 100 kg or more and received a standard dose of low-molecular-weight heparin not adjusted for weight (enoxaparin 40 mg daily). Previous studies have clearly demonstrated that weight-adjusted dosing is required to reach prophylactic anti-factor Xa levels [16]. It is therefore possible that patients in this cohort had sub-prophylactic anticoagulation. Second, in three cases, the warming catheter was left in situ for more than four days. The VTE risk of warming/cooling catheters is known to rise significantly if left in situ for longer than four days [19], and it is therefore recommended that ICY catheters not be used for longer than this time period. Third, in two cases, prophylactic anticoagulation was withheld for more than half or the entire duration of the catheter insertion. Finally, haemoconcentration is a known risk factor for VTE [20], and in two of the cases, the patient remained in a state of haemoconcentration for up to 24 h post admission to the burns unit. Furthermore, the level of hemoconcentration as evidenced by the comparative haematocrit and haemoglobin was higher in the catheter group compared to the non-catheter group and trended towards significance (*p* = 0.07 and *p* = 0.09) (Table 2). Therefore, resuscitation did not achieve normal levels of haemoconcentration at the time of catheter insertion in these cases and may have contributed to the development of VTE. Attention to the prevention of these risk factors may have further reduced the risk of VTE in this group.

## 5. Conclusions

Our study demonstrated a potentially catheter-associated VTE incidence of 10%, which is comparable to the symptomatic VTE risk for patients with equally severe burns and the VTE risk for patients with standard femoral venous catheters. Surveillance imaging such as ultrasound could be used as a precaution prior to removal of the intravascular warming catheter to diagnose VTE. Attention to potential preventable VTE risk factors by using weight-adjusted dosing of prophylactic anticoagulation, time-limited catheter use and avoidance of prolonged haemoconcentration are important strategies to minimise VTE incidence in this high-risk cohort. With these strategies, the safe use of intravascular warming catheters in a selected group of patients with major burns may be considered.

## Figures and Tables

**Table 1:** Characteristics of patients with total body surface area affected (TBSA) >20% according to insertion of intravascular warming catheter.

	Patients with Catheter (*n* = 40)	Patients without Catheter (*n* = 127)	*p*-Value
Age (years)	44 (33, 59)	41 (25, 56)	0.279
%TBSA Burn	55 (40, 65)	28 (22, 44)	<0.01 *
Mechanical Ventilation (hours)	261 (133, 600)	3 (0, 44)	<0.01 *
ICU Length of Stay (hours)	405 (274, 828)	9.8 (0, 107)	<0.01 *
Total Length of Stay (days)	62 (39, 81)	16 (3, 27)	<0.01 *
VTE	8 (20%)	5 (4%)	<0.01 *
Mortality	9 (23%)	35 (28%)	0.68

Data are median (IQR) or *n* (%; percentage). * significance = *p* < 0.05. VTE = venous thromboembolism.

**Table 2:** Characteristics of patients with total body surface area affected (TBSA) >20% with intravascular warming catheter and venous thromboembolism.

	VTE *n* = 8	No VTE *n* = 32	*p*-Value
Age (years)	49 (33, 62)	52 (31, 59)	0.883
%TBSA burn	60 (41, 69)	54 (40, 65)	0.461
Male	5 (63%)	26 (81%)	0.348
Weight (kg)	95 (70, 107)	85 (74, 96)	0.9745
ICU admission	8 (100%)	32 (100%)	>0.999
Mechanical ventilation	8 (100%)	32 (100%)	>0.999
Catheter duration (days)	4 (3, 5)	4 (3, 5.5)	0.556
Prophylactic anticoagulation administered whilst catheter in situ	6 (75%)	28 (88%)	0.580
Hct at time of catheter insertion (L/L) normal range = 0.36–0.50 L/L	0.49 (0.44, 0.57)	0.46 (0.39, 0.50)	0.07
Hb at time of catheter insertion (g/L) normal range = 128–175 g/L	161 (144, 188)	152 (133.5, 164.5)	0.09
Platelets at time of catheter insertion (×10^9^/L)	255 (199, 302)	221.5 (144.5, 243.3)	0.123
pRBC transfusion	5 (63%)	18 (56.25%)	0.709
Number of pRBC transfusions whilst catheter in situ	2 (0, 6.25)	1.5 (0, 4)	0.920
Site of VTE:			
Upper limb DVT	4 (50%)		
Lower limb DVT	1 (12.5%)		
Inferior Vena Cava DVT	1 (12.5%)		
PE	2 (25%)		
Mortality	1 (12.5%)	8 (25%)	0.655

Data are median (IQR) or *n* (%; percentage). Significance = *p* < 0.05. VTE = venous thromboembolism. PE = pulmonary embolism. DVT = deep vein thrombosis. RBC = red blood cell.

## Data Availability

Not applicable.
